# Ultrastructural Characterization of SARS Coronavirus

**DOI:** 10.3201/eid1002.030913

**Published:** 2004-02

**Authors:** Cynthia S. Goldsmith, Kathleen M. Tatti, Thomas G. Ksiazek, Pierre E. Rollin, James A. Comer, William W. Lee, Paul A. Rota, Bettina Bankamp, William J. Bellini, Sherif R. Zaki

**Affiliations:** * Centers for Disease Control and Prevention, Atlanta, Georgia, USA

**Keywords:** severe acute respiratory syndrome, SARS virus, coronavirus, electron microscopy, immunogold techniques, in situ hybridization, ultrastructure, replication complex, emerging infectious diseases

## Abstract

Severe acute respiratory syndrome (SARS) was first described during a 2002–2003 global outbreak of severe pneumonia associated with human deaths and person-to-person disease transmission. The etiologic agent was initially identified as a coronavirus by thin-section electron microscopic examination of a virus isolate. Virions were spherical, 78 nm in mean diameter, and composed of a helical nucleocapsid within an envelope with surface projections. Herein, we show that infection with the SARS-associated coronavirus resulted in distinct ultrastructural features: double-membrane vesicles, nucleocapsid inclusions, and large granular areas of cytoplasm. These three structures and the coronavirus particles were shown to be positive for viral proteins and RNA by using ultrastructural immunogold and in situ hybridization assays. In addition, ultrastructural examination of a bronchiolar lavage specimen from a SARS patient showed numerous coronavirus-infected cells with features similar to those in infected culture cells. Electron microscopic studies were critical in identifying the etiologic agent of the SARS outbreak and in guiding subsequent laboratory and epidemiologic investigations.

A large outbreak of severe pneumonia associated with human deaths occurred in late 2002 in Guangdong Province, China. Beginning in late February 2003, a similar illness was reported concurrently in Vietnam, Hong Kong, Canada, Singapore, and other countries (*1*,*2*). The disease, now known as severe acute respiratory syndrome (SARS), causes an influenzalike illness with fever, cough, dyspnea, and headache. Person-to-person transmission, combined with international travel of infected persons, accelerated the worldwide spread of the illness. By the time the outbreak was contained, 8,098 probable cases, resulting in 774 deaths, were identified in 29 countries (*3*).

A global network of 11 laboratories was established by the World Health Organization to identify the causal agent (*4*). Initial clinical and laboratory results focused on several known agents of respiratory illness, including human metapneumovirus, influenza virus, and *Chlamydia* (*4*,*5*). A virus was isolated from the oropharynx of a SARS patient and identified by morphologic characteristics as belonging to the family *Coronaviridae* (*6*–*8*); however, coronaviruses had not been a prime consideration in the differential diagnosis since they rarely cause lower respiratory tract infections in humans (*9*–*11*). Electron microscopic findings thus shifted the focus of the laboratory investigation toward verification of these observations. These findings subsequently were corroborated by immunohistochemical, immunofluorescent, and serologic assays, by additional culture isolates, and by a variety of molecular approaches, including reverse transcription–polymerase chain reaction, microarray analysis, and sequencing (*5*–*7*,*12*,*13*). As a result of those studies, the SARS-associated coronavirus (SARS-CoV) is now recognized as the etiologic agent of this syndrome.

We present here the ultrastructural features of SARS-CoV in cell culture and in a bronchial alveolar lavage (BAL) specimen. Viral immunogold labeling and ultrastructural in situ hybridization (ISH) were used to further analyze the morphogenesis of this newly emergent virus.

## Methods

Infected and uninfected Vero E6 cells were harvested 3–5 days after inoculation, inactivated by fixation and gamma irradiation (2 × 10^6^ rad), and processed for standard, immunolabeling electron microscopy (IEM) or ISH EM as previously described (*6*,*14*). For standard EM, glutaraldehyde- and osmium tetroxide–fixed specimens were embedded in Epon-substitute and Araldite (Ted Pella, Inc., Redding, CA) and sections were stained with uranyl acetate and lead citrate. Some infected and uninfected cultures were treated with 5% tannic acid solution before being embedded for standard EM (*15*). Specimens prepared for IEM and ISH assays were fixed in paraformaldehyde and glutaraldehyde and embedded in LR White resin (Ted Pella, Inc.), and sections were collected on nickel mesh grids.

A BAL specimen was obtained from a 47-year-old man within the first week of the onset of symptoms. A portion of the specimen was centrifuged at 2,000 rpm for 10 min, and the pellet was processed for standard EM.

IEM and ISH assays were performed essentially as described for Nipah virus (*14*). In brief, for IEM assays, sections were reacted with hyperimmune mouse ascitic fluid raised against SARS-CoV and then with a goat anti-mouse antibody conjugated to 12-nm colloidal gold particles (Jackson ImmunoResearch Laboratories, Inc., West Grove, PA). Negative-sense riboprobes for the ultrastructural ISH assays were prepared as previously described (*16*,*17*). Riboprobes were directed against the nucleocapsid or polymerase protein portions of the SARS-CoV genome (Table) and incorporated digoxigenin-11-dUTP. Because of the nested set structure of the coronavirus genomic RNA (genRNA) and messenger RNAs (mRNAs), the nucleocapsid riboprobe would detect all viral RNAs (*18*). Sections were reacted with a pool of nucleocapsid and polymerase probes and then with a sheep anti-digoxigenin antibody conjugated to 6-nm colloidal gold particles (Electron Microscopy Sciences, Hatfield, PA). To obtain negative controls, we performed both assays with uninfected Vero E6 cells, and infected cells were reacted with an unrelated antibody and probe for IEM and ISH procedures, respectively.

**Table:** Riboprobes used for in situ hybridization studies of SARS-CoV^a,b^

*Gene*	*Nucleotide* *positions*	*Riboprobe size* *(nucleotides)*
Polymerase	15,250–15,755	325
Nucleocapsid	29,083–29,708	625

^a^SARS-CoV, severe acute respiratory syndrome–associated coronavirus.

^b^GenBank accession no. AY27874

## Results

### Ultrastructural Characteristics of SARS-CoV–Infected Culture Cells

**Figure 1:**
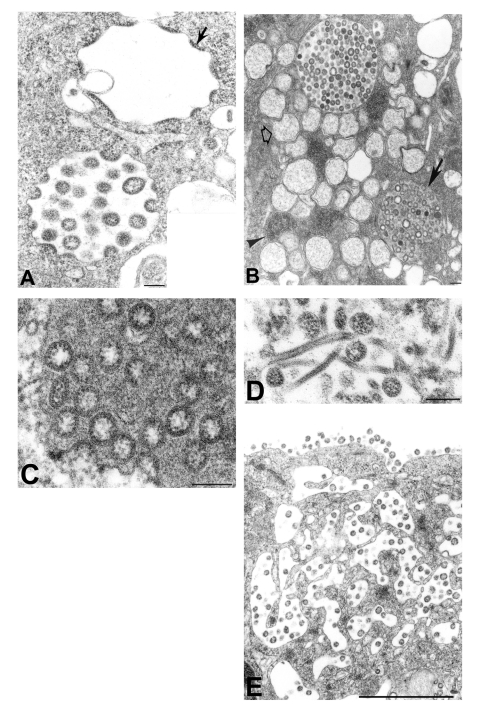
Assembly of severe acute respiratory syndrome–associated coronavirus (SARS-CoV) particles in infected Vero E6 cells. A) Apposition of nucleocapsids (arrow) along membranes of the budding compartment as particles developed and budded. Nucleocapsids measured 6 nm in diameter and were mostly seen in cross-section. Some virions had an electron-lucent center, with the nucleocapsid juxtaposed to the envelope, while others were relatively dark when the nucleocapsid was present throughout the particle. Tannic acid pre-treatment enhanced the visibility of the club-shaped viral projections (inset), which averaged 14 nm in length. B) SARS-CoV–infected cell with virus-containing vesicles, double-membrane vesicles (open arrow), and nucleocapsid inclusions (arrowhead). Note the vesicle with granular material interspersed among the virions (arrow). C) Higher magnification of a virus-containing vesicle with dark granular material. D) Tubular structures in a virus-containing vesicle. E) Virions in vesicles, which appeared to migrate toward and fuse with the plasma membrane. The characteristic lining of particles along the cell surface is seen. Bars: A, inset; B–D, 100 nm; E, 1 μm.

The morphologic features of SARS-CoV isolates were similar to those of other members of the family *Coronaviridae.* Multinucleated syncytial cells were occasionally seen. Nascent particles were formed by the juxtaposition of viral nucleocapsids along cytoplasmic membranes of the budding compartment (the membrane region between the rough endoplasmic reticulum and the Golgi complex) or occasionally on the membranes of the rough endoplasmic reticulum that form the outer layer of the nuclear membrane. Virions acquired an envelope by budding into the cisternae and formed mostly spherical, sometimes pleomorphic, particles that averaged 78 nm in diameter (Figure 1A). Cross-sections through the helical nucleocapsid were seen apposed to the viral envelope, and the interior of the particles was usually electron-lucent. Surface projections were faint in standard thin-section preparations and could be better visualized by using a tannic acid treatment (Figure 1A, inset).

Virus particles were seen in membrane-bound vesicles, either as single particles or as groups in enlarged vesicles. In some of these vesicles, dense, granular material was seen interspersed between the virions (Figure 1B,C). Tubular structures, averaging 20 nm in diameter, were seen within some virion-containing vesicles (Figure 1D). The vesicles appeared to migrate toward the cell surface and fuse with the plasma membrane, releasing the viral particles (Figure 1E). Many of the particles adhered to the plasma membrane, creating a knob-like appearance on the surface of the cells.

**Figure 2:**
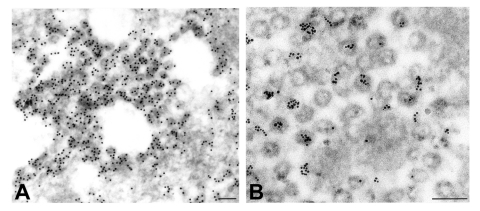
Detection of viral proteins and viral RNA associated with intracytoplasmic virions. A) Immunogold labeling of viral proteins by using hyperimmune mouse ascitic fluid directed against severe acute respiratory syndrome–associated coronavirus (12 nm gold). B) Ultrastructural in situ hybridization detection of viral RNA by using a pool of polymerase and nucleocapsid riboprobes (6 nm gold). Bars, 100 nm.

**Figure 3:**
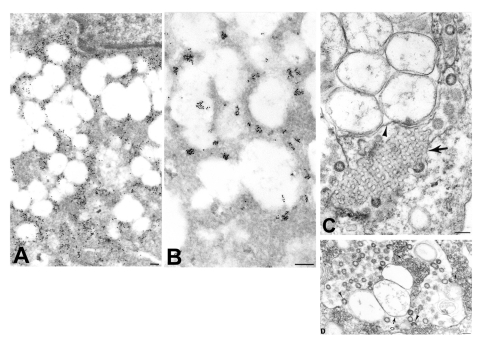
Ultrastructural characteristics of double-membrane vesicles. A) Immunogold labeling of viral proteins by using hyperimmune mouse ascitic fluid (12 nm gold) in areas of cytoplasm in close proximity to the double-membrane vesicles. B) Ultrastructural in situ hybridization detection of viral mRNA, genRNA, or both (6 nm gold) in the same areas and also at times associated with diffuse granular material within the double-membrane vesicles. C) Double-membrane vesicles showing several single-membrane vesicles enclosed within an outer membrane (arrowhead). Also present is a tubuloreticular structure (arrow) with virus particles budding from the membranes. D) Double-membrane vesicles with a large space between the inner (arrow) and outer (open arrow) membranes of the vesicles. Virions are seen budding into (arrowheads) and accumulating within the dilated inter-membrane space. At the periphery of the double-membrane vesicles are nucleocapsid inclusions; arrows point to discernable nucleocapsids (small arrows). Bars, 100 nm.

**Figure 4:**
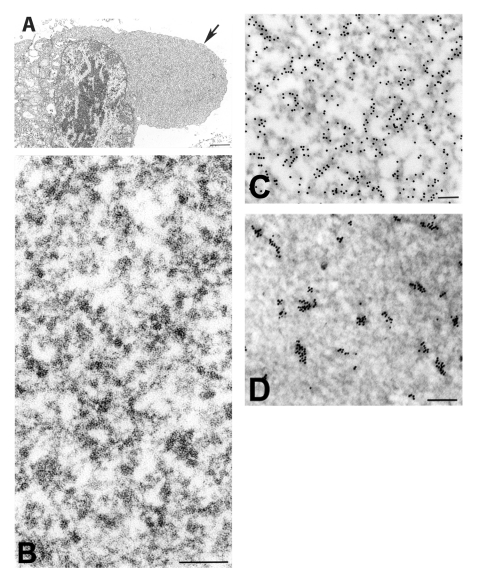
Immunogold and in situ hybridization (ISH) labeling of severe acute respiratory syndrome–associated coronavirus–infected cells. A) Cytoplasmic area that is relatively free of organelles (arrow). B) At higher magnification, these regions are shown to consist of ribosomelike and filamentous structures. Within these regions, C) viral proteins are detected by immunolabeling, using hyperimmune mouse ascitic fluid (12 nm gold), and D) ultrastructural ISH detects viral mRNA, genRNA, or both, by using a pool of riboprobes (6 nm gold). Bars, A,1 μm; B–D, 100 nm.

Viral proteins and RNA were detected in virions by IEM and ISH (Figure 2A,B), and in association with double-membrane vesicles (Figure 3A,B), nucleocapsid inclusions, and large granular areas of cytoplasm (Figure 4C,D). Double-membrane vesicles have been noted in other coronavirus-infected cells (*19*,*20*) and consist of cytoplasmic vesicles with two tightly apposed membranes (Figure 1B). In contrast, double-membrane vesicles in SARS-CoV–infected Vero E6 cells typically were composed of accumulations of multiple single-membrane vesicles enclosed within an outer membrane (Figure 3C), and virus particles were sometimes located between the two membranes (Figure 3D). Many double-membrane vesicles contained diffuse, granular material. Cytoplasmic inclusions of darkly staining viral nucleocapsids were mostly found in association with virus-containing vesicles or double-membrane vesicles (Figures 1B and 3D). Large, ill-defined areas of cytoplasm, containing ribosomelike and filamentous structures and devoid of other organelles, were noted in some SARS-CoV–infected cells (Figure 4A,B). These areas strongly labeled for viral proteins and RNA (Figure 4C,D), with IEM and ultrastructural ISH assays. No antigens or RNA were detected by reacting hyperimmune mouse ascitic fluid or riboprobes with uninfected Vero E6 cells or by reacting an unrelated hyperimmune mouse ascitic fluid or riboprobe with SARS-CoV–infected cells.

Finally, as has been reported previously for other coronaviruses, SARS-CoV–infected cells also contained tubuloreticular structures, with virions sometimes forming along the membranes (Figure 3C). The tubuloreticular structures were often found in close association with double-membrane vesicles.

### Ultrastructural Characteristics of SARS-CoV–Infected BAL Specimen

**Figure 5:**
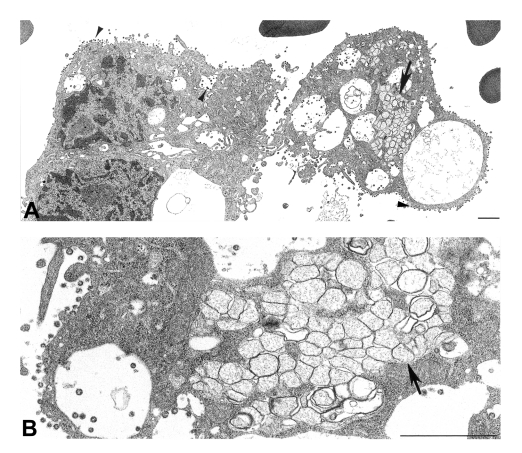
Ultrastructural characteristics of a bronchial alveolar lavage (BAL) from a patient with severe acute respiratory syndrome. A) Infected cells from a BAL specimen, showing numerous cytoplasmic and extracellular virions (arrowheads). Note the region of double-membrane vesicles (arrow), a common feature of coronavirus-infected cells. B) At higher magnification, double-membrane vesicles (arrow) are shown to contain diffuse, granular material. Bars, 1 μm.

A number of coronavirus-infected cells were seen within a BAL specimen from a SARS patient (Figure 5A,B). Virus particles budded into, and were associated with, vesicles, and extracellular virions covered the exterior surface of the cells. Areas of double-membrane vesicles containing a diffuse granular material were also seen.

## Discussion

During the global SARS outbreak of 2002 to 2003, a virus was isolated from human patients and identified by EM as belonging to the family *Coronaviridae* (*6*,*7*). Detailed studies described here on the morphogenesis of the SARS-CoV by thin-section EM found many characteristics previously described for coronaviruses (*19*,*21*,*22*). Virus particles formed upon membranes of the “budding compartment,” a term used to describe the continuous membrane system from the rough endoplasmic reticulum to the Golgi complex (*23*,*24*). Virions accumulated in dilated vesicles that appeared to migrate to the cell surface where the virus particles were released or remained adherent to the plasma membrane. Additional cytoplasmic structures associated with coronavirus infections included nucleocapsid inclusions and double-membrane vesicles, which have been proposed as the replication complex for coronaviruses (*20*), and arteriviruses (*25*), a closely related virus family that, in addition to coronaviruses, is a member of the order Nidovirales. IEM and ultrastructural ISH assays detected viral proteins and mRNA or genRNA associated with virions, double-membrane vesicles, and nucleocapsid inclusions. Coronaviruses are known to synthesize a nested set of subgenomic mRNAs (*26*), such that the nucleocapsid riboprobe used here allowed detection of all viral mRNAs in addition to genRNA. Indeed, considerable amounts of RNAs were detected in the ultrastructural ISH assays performed on SARS-CoV–infected cells.

As has been reported for other coronaviruses, additional cytoplasmic features were associated with SARS-CoV–infected cells. Tubular structures were occasionally seen within virus-containing vesicles (*27*,*28*); and cytoplasmic tubuloreticular structures, known to occur with numerous other infections (see *29*), were also found. Large granular areas of cytoplasm, relatively devoid of organelles and containing viral proteins and RNA, were noted in SARS-CoV–infected cells; such features have not been described previously for coronaviruses. While the role of these cytoplasmic areas is unclear, the close proximity of cellular ribosomes with viral proteins and RNA suggests that they may be viral translation centers. Future ultrastructural ISH and IEM studies to characterize these areas, using riboprobes and monoclonal antibodies to specific SARS-CoV genes and gene products, should help clarify this issue.

Many of these ultrastructural findings were also observed in a BAL specimen from a SARS patient (Figure 5B) (*6*). Characteristic virions in vesicles and lining the cell surface and the presence of double-membrane vesicles provided clear evidence of a coronavirus infection and suggested that viral replication was occurring in the lower airways early in the course of infection. EM examination of BAL specimens may prove to be a useful tool in the diagnosis of SARS-CoV, analogous to the use of BAL specimens to diagnose influenza infections. Recent studies have reported finding coronavirus particles in lung and gastroenteric tissues of SARS patients and experimentally infected macaques (*7*,*30*–*33*), although the viral nature of these structures has not been confirmed by IEM or ultrastructural ISH assays. Coronavirus particles may be confused morphologically with other nonviral structures routinely found in cells, including coated vesicles, multivesicular bodies, perichromatin granules, glycocalyceal bodies, and cellular projections (*29*). Therefore, a cautious approach is advisable when examining clinical specimens.

The SARS outbreak is a prime example of an emerging infectious disease that can rapidly and easily spread, reaching global proportions. With SARS, as with previous investigations of outbreaks involving such viruses as Ebola (*34*–*36*), Hendra (*37*), Nipah (*38*), and more recently, monkeypox (*39*), EM played an essential role in determining the specific virus family of the pathogen involved. In all of these cases, tissue culture amplification of a virus isolate facilitated the ultrastructural examination. Thus, traditional microbiologic and EM approaches proved pivotal in determining the etiologic agents, thereby guiding subsequent laboratory and epidemiologic investigations.
